# Full endoscopic resection of large bilateral synovial cysts in lumbar spine

**DOI:** 10.3171/2024.1.FOCVID23208

**Published:** 2024-04-01

**Authors:** Jannik Leyendecker, Nelson Sofoluke, Christoph P. Hofstetter, Sanjay Konakondla

**Affiliations:** 1 Department of Neurological Surgery, University of Washington, Seattle, Washington;; 2 Department of Orthopedics and Trauma Surgery, University of Cologne, Faculty of Medicine, Cologne, Germany; and; 3 Department of Neurosurgery, Geisinger Neuroscience Institute, Danville, Pennsylvania

**Keywords:** full endoscopic spine surgery, synovial cyst, resection, lumbar spine

## Abstract

Synovial spinal cysts cause radiculopathy and back pain, with rare reports of cauda equina syndrome. Hypermobility and instability are cornerstones for synovial cyst formation. The incidence is around 5%, and data for bilateral cysts are lacking. Surgery is indicated after conservative measures fail. Recurrence is common and is potentially due to joint violation and destabilization from open surgery. This could be prevented via ultra-minimally invasive approaches. The authors present full endoscopic removal of bilateral synovial cysts in a patient with grade 1 stable spondylolisthesis and include a 360° view for confirmation of complete decompression. Postoperatively, the patient reported immediate pain relief.

The video can be found here: https://stream.cadmore.media/r10.3171/2024.1.FOCVID23208

## Transcript

This case demonstrates the application of full endoscopic uniportal spine techniques to remove bilateral synovial cysts in the lumbar spine. I’m Sanjay Konakondla, and I’ll review the key points in the patient workup, technical nuances, and intraoperative considerations, which are all necessary for a successful surgical result.

### 0:41 Patient Presentation.

The patient is a 63-year-old male with a BMI of 41 kg/m^2^ and a complex past medical history significant for chronic kidney disease, coronary artery disease, myocardial infarction, status post stenting, diabetes, hypertension, and obesity. He presented with a > 1-year history of chronic low-back pain, weakness, and bilateral lower-extremity pain, which matched clinically with a bilateral L5 radiculopathy. On examination, the patient exhibited bilateral ankle dorsiflexion weakness graded 4+/5 bilaterally. The patient had a notable antalgic gait and used a wheelchair to mobilize due to the pain intensity. The patient failed all conservative management, which included physical therapy, pain medications, and injections.^1–4^

### 1:30 Imaging Review.

Initial imaging available for review included an MRI of the lumbar spine, which revealed transitional anatomy, lumbar spondylosis, and an L4–5 grade 1 spondylolisthesis. At the level of L4–5, bilateral synovial cysts, right greater than left, were identified with resultant significant cauda equina nerve root compression. A SPECT-CT was completed and revealed significant radiotracer uptake at the bilateral L4–5 joints. Standing neutral and flexion/extension x-rays, however, did not reveal significant dynamic instability. Due to the patient’s severe symptoms, clinical examination, and imaging findings, the patient was deemed appropriate for resection of the bilateral synovial cysts. The goal was to decompress the neural elements, halt the progression of further neurological impairment, and to improve his quality of life.^5–7^ The recommendation of a fusion procedure can also be considered in this patient with bilateral synovial cysts and advanced facet degeneration and could be supported by literature. Due to the patient’s comorbidities, stable flexion/extension x-rays, and our goals to avoid significant collateral damage, we elected to attempt only a decompression first.

### 2:41 Patient Positioning/OR Setup.

A full endoscopic surgical procedure was chosen to accomplish these goals under direct visualization. Given the patient’s medical history, our aim was to minimize surgical invasiveness, avoid instrumented fixation/fusion, and to reduce postoperative narcotic consumption. After full written and informed consent, the patient was scheduled electively for this procedure. Intraoperative positioning included an open Jackson table with a Wilson frame. The patient was induced under general endotracheal anesthesia without complication and positioned prone onto the operating table. All pressure points were accounted for and padded. Intraoperative neuromonitoring was used throughout the case. Screens were opposite the surgeon for comfort.

It is important to note that typically the surgeon and the assistant are on the same side of the pathology when the pathology is unilateral. In this case, an over-the-top approach was necessary to complete the successful resection of the bilateral synovial cysts. With this in mind, the approach was from the side of the smaller, left cyst for better off-axis view of the larger cyst when traveling contralaterally, as highlighted by the green arrows.

### 3:52 Targeting and Docking.

Intraoperative fluoroscopy is used to identify specific landmarks. After the appropriate level is identified, lines are drawn at the midline and at the disc level, which are represented by the purple dashed lines. The C-arm on the AP view is optimized to open the interspinous distance over the disc space. This distance is represented by the yellow bidirectional arrows. An initial dilator is placed at the medial edge of the inferior articulating process of L4, sequential dilators are passed, a tubular retractor is passed over the dilators, and a fluoroscopic image is completed to reveal appropriate trajectory. This is shown on the right-sided fluoroscopic image. The green circle marks the docking point, and the green arrows represent the possible trajectories traveling in between the spinous processes to reach the contralateral side. This offers the possibility of minimizing extensive bony resection when trying to reach contralaterally.

### 4:48 Introduction of the Endoscope.

The endoscope was introduced into the tubular retractor. A high-speed round course diamond tip drill was used to undermine the lamina. As any typical interlaminar endoscopic approach, the ligamentum flavum was identified and removed using graspers. The system used in this case was the joimax iLESSYS Pro set, which has a tubular retractor inner and outer diameter of 7.5 mm and 8.5 mm, respectively. This allows for the passage of an endoscope with a 4.7-mm working channel diameter and facilitates Kerrison instruments up to 4.0 mm and a round diamond drill of a 4.5-mm diameter. The drilling seen here is of the L4 inferior articulating process and the superior portion of the L5 lamina. The first sight of the contralateral larger synovial cyst can be seen and is identified by the black asterisk, and the compressed dura is represented by the blue arrow. The white asterisk represents the ipsilateral synovial cyst components.

### 5:49 Ipsilateral Cyst View.

The working channel and the endoscope are turned to identify the ipsilateral ligamentum flavum, which is removed with Kerrison punches to uncover the ipsilateral synovial cyst. The bipolar is used to release and visualize the cyst-dural plane of the ipsilateral side. This is represented by the blue arrow.

### 6:08 Developing Planes.

A ball-tipped bipolar device is used as a dissector to identify the cyst-dural plane and the full extent of the contralateral cyst. Epidural veins and bony bleeders are carefully cauterized. The ipsilateral inferior articulating process is further removed with the diamond drill bit.

### 6:31 Ipsilateral Nerve Root View.

A first glimpse of the ipsilateral traversing nerve root (identified by the black asterisk) and fat can be seen on the lateral side of the ipsilateral cyst, indicating adequate ipsilateral exposure.

### 6:45 Ipsilateral Cyst Removal.

Attention was placed on the ipsilateral cyst, and the cyst wall was removed with Kerrison punches and pituitary graspers of varying sizes. Throughout the case, the ball-tipped bipolar device was used as a dissector and to obtain hemostasis. After removal of the ipsilateral cyst, the area was inspected from top to bottom.

### 7:20 Contralateral Cyst Removal.

The contralateral cyst and the cyst wall were removed with Kerrison punches and pituitary graspers of varying sizes. Careful technique should always be emphasized to slide along the dorsal dural surface and to only travel into inviting planes. The cyst wall and ligamentum flavum ventral to the contralateral L5 superior articulating process is removed.

### 7:52 Contralateral Traversing Nerve Root View.

 After adequate cyst removal, the contralateral traversing nerve root can now be visualized (demonstrated by the white asterisk).

### 8:03 Contralateral Reach.

A now decompressed thecal sac is appreciated after removal of both cysts. The contralateral reach is again demonstrated with fluoroscopic imaging, and a curved instrument can be seen traveling dorsal to the dura to the exiting and traversing nerve root cranially and caudally, respectively.

### 8:20 Postoperative Course.

The surgery was completed without any peri- or intraoperative complications. After meticulous hemostasis, the 8-mm incision was closed with absorbable sutures and glue. Surgery time was 273 minutes. Given the bilateral nature of the pathology and difficult cyst wall to dural planes, significant time was taken to ensure maximally safe resection of the compressive cyst components. We do believe with equipment and device innovations the operative time may be reduced substantially. The patient reported immediate pain relief and was discharged home on postoperative day 1 without any issues. It should be noted that these cases are challenging, and specific consideration should be given to preoperative planning. These considerations specifically include pathology characteristics and approach trajectories. Comprehensive training in full endoscopic spine procedures, clinical experience, and surgical volume of endoscopic spine procedures are necessary for successful surgical outcomes.

## Disclosures

Dr. Hofstetter reported being a consultant for Globus, Innovasis, and Joimax; and a teacher for AO Spine and Espinea. Dr. Konakondla reported being an educational consultant for Spineology, Stryker, Joimax, and Elliquence; receiving royalties from Spineology; and receiving honoraria from Stryker, Joimax, and Elliquence.

## Author Contributions

Primary surgeon: Konakondla. Assistant surgeon: Sofoluke. Editing and drafting the video and abstract: Leyendecker, Sofoluke. Critically revising the work: Konakondla, Leyendecker, Hofstetter. Reviewed submitted version of the work: all authors. Approved the final version of the work on behalf of all authors: Konakondla. Supervision: Konakondla, Hofstetter.

## Supplemental Information

### Patient Informed Consent

The necessary patient informed consent was obtained in this study.
